# C-752-07. Rapid Enzymatic Debridement for Conflict Area Burn and Blast Injuries

**DOI:** 10.1093/jbcr/irag033.020

**Published:** 2026-04-06

**Authors:** Gefen Bloom, Yaron Shoham, Moti Harats, Josef Haik, Noa Sabag, Inbal Alkalay

**Affiliations:** Soroka University Medical Center, Be’er Sheva, HaDarom; Soroka University Medical Center, Be’er Sheva, HaDarom; Sheba Medical Center, Tel Aviv, Tel Aviv; Sheba Medical Center, Ramat Gan, HaMerkaz; Soroka University Medical Center, Be’er Sheva, HaDarom; Soroka University Medical Center, Be’er Sheva, HaDarom

## Abstract

**Introduction:**

In conflict areas and mass casualty situations limited resources restrict the availability of operating rooms (OR), prompting the need for solutions to relieve surgical bottlenecks. During the past years our medical centers dealt with a multitude of severe burns and blast injuries as a result of the ongoing conflict in our region. Many of these patients suffered from multi-trauma combined injuries without the ability to perform early eschar removal due to their general condition or lack of prolonged operating room availability due to mass casualty conditions. The aim of this study is to assess the outcomes of Bromelain Based Enzymatic Debridement (BBED) utilized for the treatment of these patients.

**Methods:**

This is an ongoing multicenter retrospective study, including 2 major medical centers situated in the conflict area. Eligible participants included burn and blast injury patients who underwent BBED for their injuries. Data collected includes patient demographics, injury characteristics, timing and setting of enzymatic debridement, pain control/anesthesia, the need for surgical interventions, and rehabilitation details.

**Results:**

During the past 2 years 166 patients have been included in the study. The average age is 28.7 ∓ 23.5 years (range 0.8-92 years) and 30% of patients are female, 70% male. The majority of patients suffered flame burns covering up to 20% TBSA (126/166 patients), however the burns area ranged up to 70% TBSA. Upper extremities were the most commonly treated area. The average time from injury to BBED application was 30 hours. None of the BBED treatments necessitated the use an OR. All BBED treatments were performed either in burn intensive care or surgical departments. Only 38% of the patients (64/166) necessitated surgical intervention following BBED, while 62% (102/166) healed spontaneously. The patient cohort also includes multi-trauma patients, suffering from a combination of blast injuries and burns, typically to upper extremities and faces, including cases of combined open hand fractures and burns that underwent BBED after fracture fixation.

**Conclusions:**

The use of BBED assisted in alleviating surgical bottlenecks in a conflict area under mass casualty conditions, allowing ORs to be used for other multi-trauma patients. In our experience its advantages include a rapid effective eschar removal without the need for an OR, feasible application under regional anesthesia in many cases, with a high rate of spontaneous healing in burns that would otherwise have necessitated surgical eschar removal and skin grafting.

**Applicability of Research to Practice:**

We found BBED an effective alternative to surgical eschar removal, especially under mass casualty conditions with limited OR availability.

**Funding for the Study:**

N/A.

